# Early Identification of Cardiovascular Adverse Events Associated With Rofecoxib Using Real‐World Data From the UK: A Nested Case–Control and Case‐Crossover Study

**DOI:** 10.1002/pds.70343

**Published:** 2026-03-09

**Authors:** Donya Moslemzadeh, Patrick C. Souverein, Svetlana V. Belitser, Eibert R. Heerdink, Olaf H. Klungel, Shahab Abtahi

**Affiliations:** ^1^ Division of Pharmacoepidemiology and Clinical Pharmacology Utrecht Institute for Pharmaceutical Sciences, Utrecht University Utrecht the Netherlands; ^2^ Research Group Innovations of Pharmaceutical Care Utrecht University of Applied Sciences Utrecht the Netherlands

**Keywords:** case‐crossover design, major adverse cardiovascular event, nested case–control design, real‐world data, rofecoxib

## Abstract

**Background:**

Traditional pharmacovigilance systems have limitations in detecting common adverse drug reactions. We investigated whether real‐world data (RWD) could have detected rofecoxib's cardiovascular adverse effects earlier using nested case–control (NCC) and case‐crossover (CCO) designs.

**Methods:**

We included adult rofecoxib users from the UK CPRD GOLD (1999–2004). In NCC design, cases of a first major adverse cardiovascular event (MACE) were matched with four controls on age, sex, practice and calendar time. Rofecoxib exposure was categorised as current (≤ 3 months), recent (3–6), or past use (> 6) in NCC, and assessed at the start of each 3‐month interval in CCO design. Exposure odds in CCO were compared between a 3‐month risk with four reference windows. Conditional logistic regression models estimated adjusted intensity ratio (aIR). To identify the shortest time necessary to detect the association, analyses were conducted in 1‐, 2‐, 3‐, 4‐ and 5‐years after the drug's market uptake.

**Results:**

Three thousand two hundred and eighteen cases were matched to 10 745 controls (mean age 73.8 years, 66% female). In NCC, current rofecoxib use (42% of cases) was associated with an 18% higher risk of MACE (aIR 1.18, 95% CI 1.08–1.29) versus past use. The CCO (3210 risk and 12 737 reference windows) showed an 83% increased risk of MACE (aIR 1.83, 95% CI 1.53–2.18). First signal emerged after 2 years with CCO (aIR 3.94, 95% CI 1.88–8.25), and after 3 years with NCC design (aIR 1.46, 95% CI 1.18–1.81).

**Conclusion:**

Using RWD, cardiovascular adverse effects of rofecoxib could have been detected within 2 years of the market entry in the UK, well before traditional pharmacovigilance methods. This supports incorporating RWD analysis into routine drug safety monitoring.

## Key Points

- Using real‐world data from the UK, rofecoxib's cardiovascular adverse events were detected 2 years after market uptake, well before traditional pharmacovigilance methods.
- Both nested case–control (NCC) and case‐crossover (CCO) identified the increased cardiovascular risks of rofecoxib, showcasing their applicability for signal detection.
- NCC is computationally light, needs few cases, and when restricted to ever‐users, can minimise confounding by indication.
- CCO self‐matches cases, cancels all time‐fixed patient factors, and is ideal when exposure is transient and outcome is acute.
- Real‐world data and pharmacoepidemiologic methods can be integrated into routine drug safety monitoring in detecting adverse events associated with new drugs.

## Plain Language Summary

Non‐steroidal anti‐inflammatory drugs (NSAIDs) are common painkillers that ease pain and inflammation but may cause stomach ulcers. A newer group, COX‐2 inhibitors, were developed with fewer stomach side effects. However, rofecoxib, as a COX‐2 inhibitor, later showed a higher risk of heart attack than naproxen (an older NSAID). It took the manufacturer 5 years to withdraw rofecoxib from the market due to higher risks of heart attacks and strokes. Historically, detecting adverse drug reactions (ADRs) of a new medication relies on spontaneous reporting systems (SRSs). However, this has limitations such as difficulty in detecting a common event. In the case of rofecoxib, SRS could not detect cardiovascular events earlier, and the drug was withdrawn after findings from post‐authorisation clinical trials. Nowadays, with increasing access to real‐world data (RWD), various studies can be conducted to investigate the safety profile of medications. Using RWD, we showed that rofecoxib's increased risk of heart attack, stroke, and heart failure could have been detected only 2 years after the drug's market uptake in the UK, 3 years sooner than its official withdrawal. The improved signal detection methodologies based on RWD can benefit patient care and policy making for swifter detection of ADRs in real patient groups.

## 1 Introduction

Rofecoxib, a selective COX‐2 inhibitor, was withdrawn from the market in 2004 due to cardiovascular adverse events [1]. During its marketing period (May 1999–September 2004), over 80 million patients used rofecoxib worldwide [2]. The Adenomatous Polyp Prevention on Vioxx (APPROVe) trial [3, 4], revealed 16 excess myocardial infarction (MI) or stroke events per 1000 patients without cardiovascular disease history [2], potentially translating to 1.3 million additional major adverse cardiovascular events (MACE) globally during rofecoxib's marketing period.

Approximately 4% of newly introduced drugs are eventually withdrawn from the market due to serious adverse effects not detected during pre‐marketing trials [5]. While pre‐authorisation clinical trials aim to identify potential risks, certain adverse reactions only emerge when medications are used in larger, more diverse populations over extended periods. This highlights the importance of effective post‐market surveillance. Current pharmacovigilance primarily relies on post‐authorisation spontaneous reporting systems (SRSs), where patients and healthcare professionals report suspected adverse events to pharmacovigilance centres. However, SRSs have significant limitations including substantial underreporting (estimated at > 90% for serious adverse drug reactions, ADRs), inability to detect delayed adverse events, and poor detection of common events in general populations, such as cardiovascular events [6, 7, 8]. Moreover, post‐authorisation randomised controlled trials (RCTs) are quite expensive, lengthy, and usually contain a relatively small study population, which hampers their use for swift detection of an ADR. Perhaps a combination of these limitations of SRSs and post‐authorisation RCTs led to such delayed withdrawal of rofecoxib from the market.

The emergence of real‐world data (RWD), coupled with advances in IT infrastructure, improved data quality, and refined pharmacoepidemiological methods, offers new opportunities for drug safety monitoring [9]. Electronic health records (EHRs) and administrative or claims databases provide longitudinal information on diverse populations, making them valuable for evidence generation for regulatory use [10]. RWD studies can vary from traditional pharmacoepidemiologic approaches such as case–control design to more novel methods such as case‐only designs [11].

This study aimed to assess whether cardiovascular adverse effects of rofecoxib could have been detected earlier using RWD from the UK. We employed both a nested case–control (NCC) and case‐crossover (CCO) study to evaluate the association between rofecoxib and MACE at different time windows after drug marketing. By comparing our findings with those from pivotal RCTs, we sought to validate the utility of RWD for early adverse event detection.

## 2 Methods

### 2.1 Data Source

This study used data from the Clinical Practice Research Datalink (CPRD) GOLD, one of the world's largest primary care databases. The CPRD contains anonymised medical records from the UK's general practitioners (GPs) [12], covering over 21.5 million total patients with acceptable data for research purposes as of December 2024 [13]. The database comprises comprehensive patients' information, including demographics, symptoms, diagnoses, prescriptions, vaccination history, laboratory results, and referrals to hospital and specialist care (secondary care) since 1987 with demonstrated high validity for cardiovascular outcomes [14, 15].

### 2.2 Study Population

The study population (i.e., base cohort) comprised adult patients (≥ 18 years old) with at least one rofecoxib prescription between 20 May 1999 (UK market entry) and 30 September 2004 (withdrawal). The cohort entry date was defined as the date of the first rofecoxib prescription. All patients were required to have at least 1 year of pre‐cohort history to ensure complete medical records and valid registration status throughout the study period.

### 2.3 Exposure and Outcome

The primary outcome was the first MACE defined as MI, stroke, and heart failure (HF) [16]. These outcomes were identified using validated Read‐codes in CPRD. The index date was defined as the date of outcome occurrence. To capture incident cases only, we excluded individuals with any recorded MACE before cohort entry.

The exposure of interest was rofecoxib prescription which was retrieved using CPRD product codes. Exposure to rofecoxib was stratified based on the most recent prescription before the index date into current (≤ 3 months), recent (3–6 months), and past use (> 6 months) for the NCC and was assessed at the start of each 3‐month interval for the CCO design. Information on dose and treatment duration was mapped using prescribed daily dose (dosageid), number of tablets (qty), and strength from the Therapy file and CPRD GOLD's Products dictionary.

We adopted the two NCC and CCO designs in this case‐study because both are recognised tools for post‐authorisation safety monitoring [17]. The NCC is the standard design for rare outcomes, needs far less person‐time, and is computationally lighter and faster to run than a cohort. Also by sampling controls only from ever‐users, it improves baseline comparability and reduces confounding by indication [18, 19]. The CCO complements this by comparing each case's exposure immediately before the event with the same person's earlier records, cancelling all time‐invariant confounders. It is preferred when the outcome is acute and the exposure is transient [20], as was the case of rofecoxib and sudden cardiovascular events.

### 2.4 Nested Case–Control (NCC) Design

**FIGURE 1:**
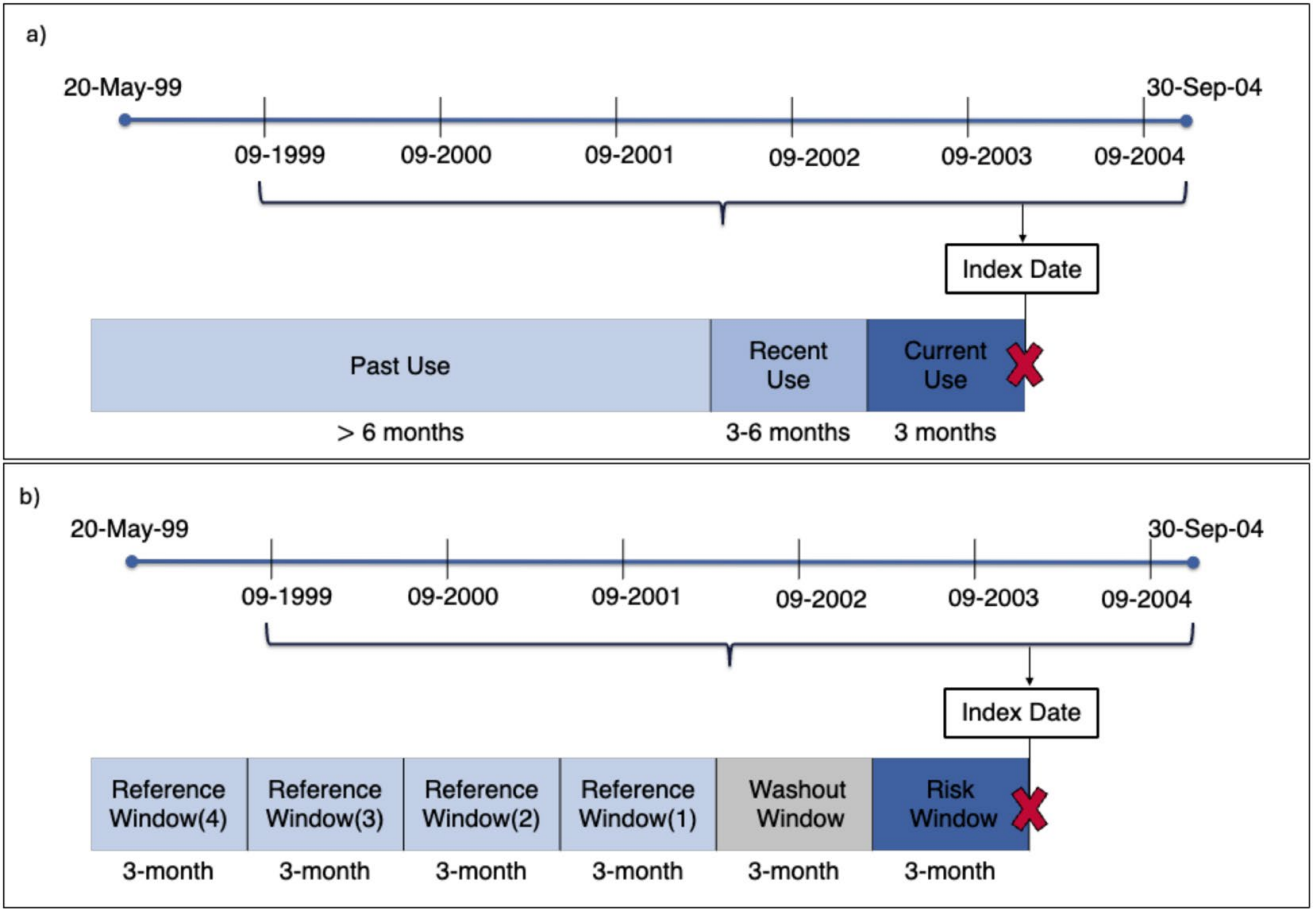
Nested case–control and case‐crossover design. The index date is the date of the first occurrence of the outcome. (a) Rofecoxib exposure was classified based on the most recent prescription before the index date into current (≤ 3 months), recent (3–6 months), and past use (> 6 months). (b) Only among the cases, we defined a 3‐month risk window before the index date, a 3‐month washout window before that, and up to four prior 3‐month reference windows.

Cases were defined as patients who experienced first MACE during study period (Figure 1). For each case, up to four controls were matched on age, sex, GP practice, and calendar time (±30 days). The index date for controls was the same date as that of the corresponding case.

In secondary analyses, we examined dose–response and duration‐response relationships among current users, by categorising daily doses (< 25 mg and ≥ 25 mg) and duration of continuous use (≤ 1 month, 1–3 months, 3–6 months and > 6 months) [21, 22], using the method explained previously [23, 24]. Here, if the daily dose value was missing or zero, it was assumed to be ‘as needed (pro‐re‐nata, PRN)’ and replaced by 0.5. The missing or zero value for the duration was replaced by the median duration of use (i.e., 28 days), and implausibly high values were replaced by the 3rd interquartile range (i.e., 112 days).

We conducted subgroup analysis stratified by sex, age groups (18–49, 50–64, 65–79, ≥ 80), main indications for rofecoxib use (rheumatoid arthritis (RA), back pain, osteoarthritis (OA)), and history of cardiovascular disease (post hoc analysis).

### 2.5 Case‐Crossover (CCO) Design

In this second design, only the cases were included, and their past experience served as their own control. In this CCO design, we defined ‘risk window’, which entailed the last 3 months before the index date and up to four ‘reference windows’, which were 3‐month periods in the past, and by definition at least 3‐months apart from the risk window with a ‘washout window’ (Figure 1). The reference windows must be between May 1999 and Sep 2004 when the drug was on the market. Moreover, only cases with an index date equal to or above 9 months (≥ 273 days) after the drug's market approval were included so every case had at least one reference window (i.e., a 91‐days reference +91‐days washout +91‐days risk windows).

As a sensitivity analysis, we repeated the analyses with a risk and reference windows of 1‐month and 6‐months, but the washout window remained at 3‐months to not mix the exposure classification between the risk and reference windows. Cases were included if they had at least one reference window.

### 2.6 Cumulative Uptake Time Analysis

Additionally, we conducted a cumulative uptake time analysis in both NCC and CCO designs. The idea was to identify the shortest time necessary to detect the association between rofecoxib use and MACE since the drug was initially introduced to the market, considering sufficient sample size and the plausible biological mechanism behind. To do that, we classified our study period into the following time windows: 1‐, 2‐, 3‐, 4‐ and 5‐years after the market uptake time (20 May 1999).

### 2.7 Potential Confounders

Potential confounders were identified based on established risk factors for cardiovascular events and clinical relevance. Age, sex, GP practice and calendar time were addressed through matching. The most recent information on smoking, and body mass index (BMI) was determined at the index date. To account for heathy‐use and surveillance biases, we assessed healthcare utilisation by measuring the frequency of GP contacts in the year preceding the index date. A history of the following comorbidities before the index date was assessed: alcohol abuse, back pain, cardiovascular diseases, chronic kidney disease (CKD), chronic liver disease, chronic respiratory diseases, diabetes (type 2), dysmenorrhea, hyperlipidaemia, hypertension, malignant neoplasms (excluding non‐melanoma skin cancers), RA, OA and migraine. Additionally, the use of comedications in the 3‐months prior to the index date was considered: angiotensin‐converting enzyme inhibitors and angiotensin receptor blockers, aspirin, beta‐blockers, calcium channel blockers, corticosteroids, diuretics, glycosides (e.g., digoxin), hormone replacement therapy, immunosuppressants (other than corticosteroids), lipid‐lowering agents (e.g., statins, fibrates, nicotinic acid or niacin, ezetimibe), nitrates, NSAIDs (other than rofecoxib), platelet aggregation inhibitors and vasodilators.

### 2.8 Statistical Analysis

Baseline characteristics were summarised using descriptive statistics, with continuous variables presented as means (±SD) and categorical variables as frequencies and proportions. Conditional logistic regression models were fitted in both NCC and CCO analyses to estimate the association between rofecoxib use and MACE (using the SAS PHREG procedure). Following the multiplicative intensity model proposed by Aalen [25, 26], we estimated intensity ratios (IRs) with 95% confidence intervals. In the NCC regression model, past use was considered as a reference for comparison. The final set of confounders in the conditional logistic regression model of the NCC design was selected after checking in univariate models (using the change‐in‐estimate criterion with a 10% cut‐off) [27], and broadened based on experts' opinion and subject matter knowledge due to the screening nature of the study. In the CCO study, age was included in the model as the only time‐varying confounder. Collinearity between potential confounders was assessed, and if detected, comorbidities were prioritised over comedications. Data were analysed using SAS version 9.4.

## 3 Results

**FIGURE 2:**
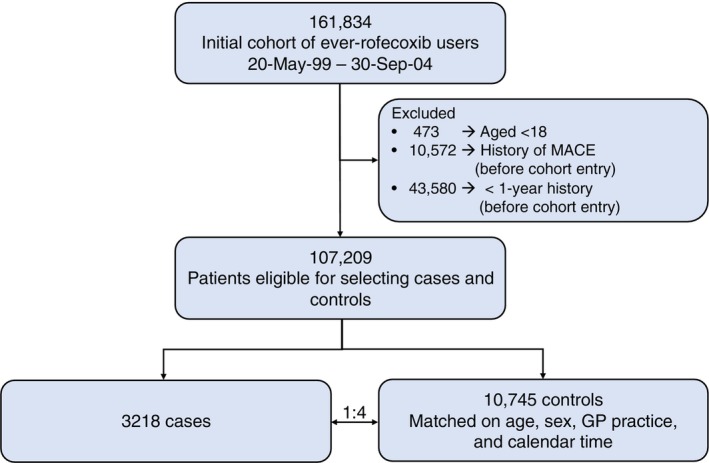
Flowchart of the establishment of the study population.

We identified 3218 cases with first MACE between 1999 and 2004, who were matched with 10 745 controls (Figure 2). The mean age was 74 years (±11 years) among cases and 66% were women (Table 1). Cases exhibited a higher burden of cardiovascular risk factors compared to controls, including higher prevalence of cardiovascular diseases (43% vs. 29%), hypertension (42% vs. 36%), and diabetes (13% vs. 8%). In the 3‐months preceding the index date, cases more frequently used medications, particularly aspirin (30% vs. 18%), diuretics (43% vs. 32%), and corticosteroids (10% vs. 7%).

**TABLE 1:** Baseline characteristics of the study population of rofecoxib users in the UK between 1999–2004.

Characteristics	Cases (% or ±SD)	Controls (% or ±SD)
Total number	3218	10 745
Sex		
Men	1103 (34.3%)	3444 (32.1%)
Women	2115 (65.7%)	7301 (67.0%)
Mean age in years (±SD)	73.8 (±10.8)	72.9 (±10.4)
Age groups		
18–49	83 (2.6%)	271 (2.5%)
50–64	527 (16.4%)	1951 (18.2%)
65–79	1506 (46.8%)	5336 (49.7%)
80+	1102 (34.2%)	3187 (29.7%)
Mean BMI (±SD) (at index date)	27.7 (±5.5)	27.3 (±5.3)
BMI categories		
< 20.0	156 (4.9%)	485 (4.5%)
20.0–24.9	777 (24.2%)	2834 (26.4%)
25.0–29.9	1081 (33.6%)	3833 (35.7%)
30.0–34.9	565 (17.6%)	1811 (16.9%)
≥ 35.0	281 (8.7%)	741 (6.9%)
Missing	358 (11.1%)	1041 (9.7%)
Smoking status (at index date)		
Never	1170 (36.4%)	4449 (41.4%)
Current	511 (15.9%)	1580 (14.7%)
Former	1446 (44.9%)	4471 (41.6%)
Missing	91 (2.8%)	245 (2.3%)
Healthcare utilisation (±SD)^a^	15 (±12.2)	12 (±9.5)
Main indications		
Backpain	1659 (51.6%)	5234 (48.7%)
Osteoarthritis	2271 (70.6%)	7309 (68.0%)
Rheumatoid arthritis	231 (7.2%)	601 (5.6%)
Comorbidities (ever before index date)		
Alcohol abuse	83 (2.6%)	229 (2.1%)
Cardiovascular disease^b^	1395 (43.4%)	3099 (28.8%)
Chronic kidney disease	92 (2.9%)	177 (1.7%)
Chronic liver disease	14 (0.4%)	26 (0.2%)
Chronic respiratory disease	559 (17.4%)	1649 (15.4%)
Diabetes (type 2)	410 (12.7%)	814 (7.6%)
Dysmenorrhea	19 (0.6%)	75 (0.7%)
Hyperlipidaemia	363 (11.3%)	926 (8.6%)
Hypertension	1350 (42.0%)	3878 (36.1%)
Malignancies (excluding non‐melanoma skin cancers)	417 (13.0%)	1382 (12.9%)
Migraine	195 (6.1%)	576 (5.4%)
Co‐medications (3 months before index date)		
ACE inhibitors and ARBs	890 (27.7%)	1894 (17.6%)
Aspirin	948 (29.5%)	1927 (17.9%)
Beta blockers	640 (19.9%)	1733 (16.1%)
Calcium channel blockers	625 (19.4%)	1664 (15.5%)
Corticosteroids	334 (10.4%)	755 (7.0%)
Digoxin and other glycosides	158 (4.9%)	195 (1.8%)
Diuretics	1392 (43.3%)	3399 (31.6%)
HRT	129 (4.0%)	470 (4.4%)
Immunosuppressants^c^	78 (2.4%)	170 (1.6%)
Lipid lowering agents^d^	621 (19.3%)	1389 (12.9%)
Nitrates	397 (12.3%)	662 (6.2%)
NSAIDs (other than rofecoxib)	699 (21.7%)	2400 (22.3%)
Platelet aggregation inhibitors	172 (5.3%)	161 (1.5%)
Vasodilators	6 (0.2%)	8 (0.1%)

Abbreviations: ACE, angiotensin converting enzyme; ARBs, angiotensin receptor blockers; BMI, body mass index; HRT, hormone replacement therapy; NSAID, non‐steroidal anti‐inflammatory drugs.

^a^ Frequency of GP visit in 1‐year before index date.

^b^ Cardiovascular diseases include a history of ischaemic heart disease, cardiac arrhythmias, cardiac myopathies, peripheral arterial diseases, arterial embolism and thrombosis, valvular diseases, myocarditis and pericarditis.

^c^ Immunosuppressants include disease‐modifying antirheumatic drugs (DMARDs).

^d^ Lipid lowering agent include statins, fibrates, ezetimibe and nicotinic acid.

### 3.1 NCC Analysis

**FIGURE 3:**
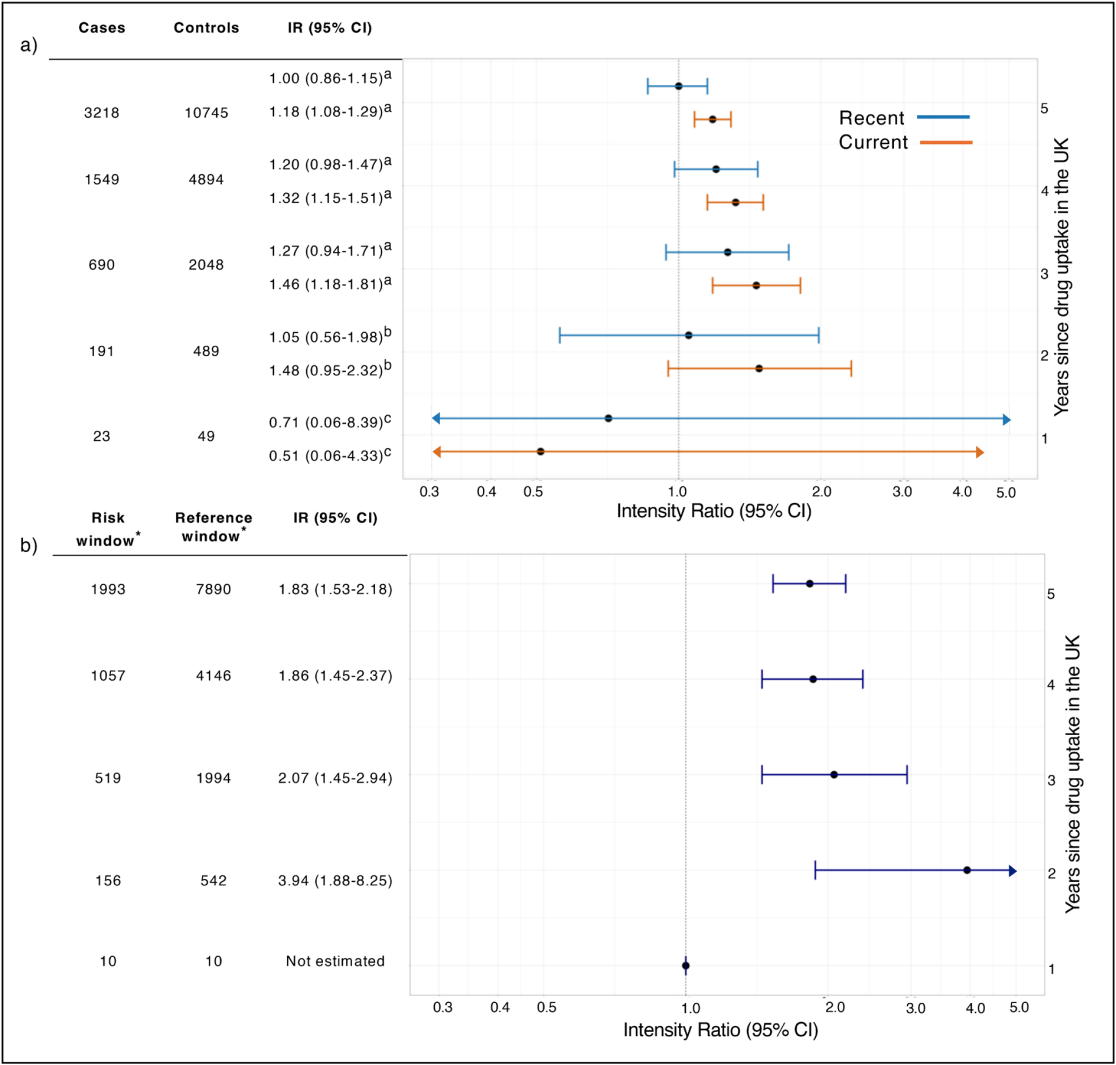
Results of the cumulative uptake time analysis from: (a) the nested case–control and (b) and case‐crossover designs. ^a^Adjusted for smoking status, BMI, alcohol abuse, RA, OA, back pain, migraine, cardiovascular disease, hyperlipidaemia, hypertension, chronic liver disease, chronic kidney disease, diabetes, malignant neoplasms, corticosteroids, platelet aggregation inhibitors, NSAIDs and healthcare utilisation. ^b^Adjusted for smoking status, BMI, RA, OA, back pain, migraine, cardiovascular disease, hyperlipidaemia, hypertension, diabetes, malignant neoplasms, corticosteroids, NSAIDs and healthcare utilisation. ^c^Adjusted for smoking status, cardiovascular disease and diabetes. *Number of discordant risk and reference windows was reported in the case‐crossover graph.

Among the cases, 1350 (42%) were current users of rofecoxib, 335 (10%) were recent users and the remaining 1533 (48%) were past users. Current use of rofecoxib was associated with a 19% increased risk of MACE compared to past use (adjusted IR [aIR] 1.18, 95% CI 1.08–1.29), while recent use showed no significant association (aIR 1.00, 95% CI 0.86–1.15) (Table 2). The cumulative uptake time analysis revealed that this association became statistically significant only after 3 years of market availability (year 3‐aIR 1.46, 95% CI 1.18–1.81), with no significant associations earlier (year 1‐aIR 0.51, 95% CI 0.06–4.33, year 2‐aIR 1.48, 95% CI 0.95–2.32) (Figure 3).

**TABLE 2:** Results of the nested case–control design of the association between rofecoxib and major cardiovascular adverse events in the UK, including the main analysis, and dose‐ and duration‐response relationships.

	MACE cases (% total)	Controls (% total)	Crude IR (95% CI)	Adjusted IR^a^ (95% CI)
Total	3218	10 745		
Rofecoxib exposure				
Past use	1533 (47.6%)	5532 (51.5%)	Reference	
Current use	1350 (42.0%)	4025 (37.5%)	1.18 (1.08–1.29)	1.18 (1.08–1.29)
Recent use	335 (10.4%)	1188 (11.1%)	1.01 (0.88–1.15)	1.00 (0.86–1.15)
Dose–response (among current users)				
< 25 mg	768 (23.9%)	2267 (21.1%)	1.16 (1.05–1.29)	1.19 (1.06–1.32)
≥ 25 mg	582 (18.1%)	1758 (16.4%)	1.21 (1.08–1.36)	1.17 (1.04–1.32)
Duration‐response (among current users^b^)				
≤ 1 month	397 (12.3%)	1376 (12.8%)	1.02 (0.90–1.17)	1.05 (0.91–1.20)
1–3 months	319 (9.9%)	1015 (9.5%)	1.09 (0.95–1.26)	1.12 (0.96–1.30)
3–6 months	247 (7.7%)	646 (6.0%)	1.34 (1.14–1.57)	1.28 (1.08–1.52)
> 6 months	542 (16.8%)	1514 (14.1%)	1.27 (1.13–1.43)	1.25 (1.11–1.42)

Abbreviations: CI, confidence interval; IR, intensity ratio.

^a^ Adjusted for smoking status, BMI, alcohol abuse, RA, OA, back pain, migraine, cardiovascular disease, hyperlipidaemia, hypertension, chronic liver disease, chronic kidney disease, diabetes, malignant neoplasms, corticosteroids, platelet aggregation inhibitors, NSAIDs and healthcare utilisation.

^b^ Total number of current users here among cases and controls is different from the models above, as this was calculated by constructing treatment episodes based on prescription dates, number of pills prescribed, and dosing regimen (see Methods), instead of relying only on prescription dates.

Secondary analyses demonstrated similar risk elevations for both current high (≥ 25 mg: aIR 1.17, 95% CI 1.04–1.32) and low‐dose rofecoxib use (< 25 mg: aIR 1.19, 95% CI 1.06–1.32) (Table 2). Duration‐response analysis showed increased risk only with continuous use exceeding 3 months (3–6 months: aIR 1.28, 95% CI 1.08–1.52, and > 6 months: aIR 1.25, 95% CI 1.11–1.42).

Subgroup analyses revealed a numerically stronger association among men (current use: aIR 1.26, 95% CI 1.07–1.48) compared to women (current use: aIR 1.14, 95% CI 1.02–1.28). Among indication‐specific analyses, a positive association was observed in patients with back pain (aIR 1.30, 95% CI 1.11–1.51) and OA (aIR 1.19, 95% CI 1.06–1.34), while the association in RA patients was statistically nonsignificant. Additionally, current use of rofecoxib only in patients with no history of cardiovascular disease had a significant association with MACE (aIR 1.23, 95% CI 1.18–1.41) (Table S1).

### 3.2 CCO Analysis

The CCO analysis included 3210 risk windows matched to 12 737 reference windows (Table 3). Of 15 947 total risk and reference windows, 9883 (62%) were included as discordant sets in the analysis (1993 risk windows–50% exposed, 7890 reference windows–30% exposed). The overall association between rofecoxib use and MACE was stronger in the CCO design (aIR 1.83, 95% CI 1.53–2.18) compared to the NCC design. The cumulative uptake time analysis detected a significant association 2 years after market entry in the UK (aIR of 3.94, 95% CI 1.88–8.25), but not earlier (Figure 3).

**TABLE 3:** Results of the case‐crossover design of the association between rofecoxib and major cardiovascular adverse events in the UK and sensitivity analyses.

Rofecoxib exposure windows	Risk window		Reference windows		Adjusted IR^a^ (95% CI)
	Total	Exposed	Total	Exposed	
Main analysis					
3‐month	3210	1344 (41.9%)	12 737	3700 (29.1%)	1.83 (1.53–2.18)
Sensitivity analyses					
1‐month	3218	825 (25.6%)	12 858	2746 (21.4%)	1.46 (1.26–1.71)
6‐month	3180	1650 (51.9%)	11 915	3324 (27.1%)	1.83 (1.44–2.32)

Abbreviations: CI, confidence interval; IR, intensity ratio.

^a^ Adjusted for age.

The sensitivity analysis assuming risk and reference windows of 1‐month yielded a 46% increased risk of MACE (aIR 1.46, 95% CI 1.26–1.71), while with exposure windows of 6‐months long there was an 83% increased risk of MACE (aIR 1.83, 95% CI 1.44–2.32) (Table 3).

## 4 Discussion

Using RWD from the UK, we investigated the earliest possible detection of cardiovascular adverse effects associated with rofecoxib after its market introduction. The NCC design identified a 18% increased risk of MACE among current users compared to past users, with the association becoming statistically significant 3 years after market entry. The CCO design detected a stronger association, that is, 83% increased risk, and identified the signal 2 years after market introduction, suggesting potential advantages of self‐controlled designs for early safety signal detection.

Our findings align with evidence from pivotal RCTs, though with varying effect magnitudes. The VIGOR trial [22], first documented this association, reporting a 2.4‐times increased risk of MI in rofecoxib users compared to naproxen users. The APPROVe trial, comparing rofecoxib to placebo [3], found a 1.9‐fold increased risk of cardiovascular events (a broader definition than ours), more closely aligned with our CCO estimates. The differing effect sizes likely reflect variations in outcome definitions, comparison groups, study populations, and unmeasured confounding in our study.

Our NCC results are partially consistent with earlier observational studies. The case–control study by Andersohn et al. using the CPRD data found an OR of 1.29 (CI 1.02–1.63) for MI with current use of rofecoxib compared to non‐use [28]. While their effect estimate aligns with our NCC findings (aIR 1.18), the slight difference might be explained by varying eligibility criteria applied (age, history of MI, etc.), outcome definitions (MI vs. MACE), and reference groups (non‐use instead of past use). Noteworthy, Andersohn et al. looked into drug‐class safety profiling across several COX‐2 inhibitors and non‐selective NSAIDs, while our intention was to use the ‘rofecoxib case‐study’ to assess how a RWD study can perform for early signal detection. While we found comparable associations with high‐ (aIR 1.17) and low‐ doses (aIR 1.19), both lying very close to our main result (aIR 1.18), Andersohn et al. and Graham et al. found a pronounced dose–response relationship [21, 28].

Regarding methods for safety monitoring of newly marketed drugs, two recent studies investigated the potential role of RWD and pharmacoepidemiologic methodologies using rofecoxib and cardiovascular events as an example. Patadia and colleagues, implemented a hypothesis‐free signal detection method to explore the association comparing seven European healthcare databases (EU‐ADR project) with WHO‐VigiBase [29]. Using the longitudinal gamma Poisson shrinker method, they detected the MI signal of rofecoxib 4 years earlier than SRS. Their findings highlight the limitation of SRS in detecting common adverse effects and the role of RWD in safety monitoring. Moreover, a recent case‐time control and cohort study within Danish healthcare registers found that the association between rofecoxib and MI could have been identified as early as 1.5 years with a case‐time control design and 3.5 years with a cohort design [30]. The results of this study and ours are aligned in the sense of suggesting that a case‐only design might be superior than a traditional one (i.e., cohort or case–control) for drug safety monitoring purposes. Using only those subset of patients who experienced the ADR of interest, the case‐only designs are quick, efficient (especially for an acute outcome and transient exposure), and can reliably contain the residual confounding and selection bias by comparisons to past history of same patients [31]. The early detection of ADRs through RWD could have significant clinical implications, in terms of awareness, and potentially preventing those through earlier intervention or discontinuation. Also, regulatory bodies can benefit by having swifter responses to emerging safety concerns of new medications [10, 17].

The current study had several strengths. The use of CPRD GOLD provided a large, representative sample of rofecoxib users with comprehensive clinical information. The use of two study designs offered insights into methodological considerations for safety surveillance. Despite matching and statistical adjustment in the NCC, and inherent adjustment of time‐invarying confounders in the CCO design, residual confounders could have affected the observed associations. Both the exposure and outcome of interest of this study are suitable candidates for a CCO design [20]. Rofecoxib was an analgesic, so patients typically took it only when they experienced pain. Also, MI and stroke were acute outcomes with a sudden onset. The only exception here was heart failure. To prevent a potential reverse causality [32], and buffering issues with adherence and delays with dispensing time, we defined the current use and risk windows as 3‐months rather than 1‐month. Moreover, through CPRD we could include important lifestyle parameters such as smoking, BMI, and alcohol abuse as risk factors for cardiovascular morbidity.

However, several limitations warrant discussion. Despite CPRD's validated recording of cardiovascular outcomes, potential misclassification of heart failure might have affected our estimates. Unlike well‐recorded MI and stroke [15], the number of heart failure events might have not been adequately captured in the primary care database which might underestimate the association. Moreover, the assumption of low‐dose use for ‘as needed’ prescriptions could have introduced exposure misclassification. The weaker associations observed with shorter risk windows might reflect both exposure misclassification and immeasurable time bias due to lack of drug use data during hospitalisations [33]. Furthermore, CPRD lacks information on over‐the‐counter medications (e.g., aspirin and other NSAIDs). As we included other NSAIDs use as a covariate in our final model, an incomplete capture of its actual use might have led to some unmeasured confounding, but there is no evidence that this was different among cases and controls. Finally, case‐crossover design is sensitive to prescribing trends: rising use inflates the risk estimate, whereas the drop after the 2002 label change might have pushed it towards the null [2].

## 5 Conclusion

This study demonstrated that cardiovascular adverse effects of rofecoxib could have been detected within 2 years of market entry using RWD and appropriate pharmacoepidemiologic methodologies, considerably earlier than traditional pharmacovigilance systems or post‐authorisation clinical trials. Particularly, the use of case‐only designs, such as CCO, showed promise for early signal detection. These findings advocate for the systematic integration of RWD analysis into routine drug safety monitoring systems for quicker signal detection and more timely regulatory action.

## Author Contributions

D.M., P.C.S. and S.A. had full access to all the data in the study and take responsibility for the integrity of data and accuracy of data analysis. All authors were responsible for the concept and design of the study. P.C.S. conducted data acquisition. P.C.S., D.M., S.A. and S.B. were in charge of data analysis. All authors were involved in the interpretation of results. D.M. wrote the first draft of the manuscript, and all authors critically revised the manuscript for important intellectual content. P.C.S. and S.A. were responsible for administrative, technical or material support. S.A. and O.H.K. have supervised the lead author (D.M.) in conducting this study.

## Funding

The authors have nothing to report.

## Disclosure

The authors have nothing to report.

## Ethics Statement

This study was reviewed and approved by Research Data Governance (RDG) Process (reference 23_003111), which is responsible for reviewing protocols for scientific quality. This study is based in part on data from the Clinical Practice Research Datalink obtained under licence from the UK Medicines and Healthcare products Regulatory Agency. The data is provided by patients and collected by the NHS as part of their care and support. The interpretation and conclusions contained in this study are those of the authors alone.

## Consent

The authors have nothing to report.

## Conflicts of Interest

The authors declare no conflicts of interest.

## Supporting information


**Table S1:** Subgroup analysis in the nested case‐control design of the association between rofecoxib and major cardiovascular adverse events in the UK.

## Data Availability

Patient level data used in this study were obtained through a multi‐study institutional license and are not publicly available. Details on how to apply for data access can be found at https://cprd.com/data‐access.

## References

[1] Merck , Merck Announces Voluntary Worldwide Withdrawal of VIOXX (Merck, 2004).

[2] E. J. Topol , “Failing the Public Health—Rofecoxib, Merck, and the FDA,” New England Journal of Medicine 351, no. 17 (2004): 1707–1709, 10.1056/nejmp048286. 15470193

[3] R. S. Bresalier , R. S. Sandler , H. Quan , et al., “Cardiovascular Events Associated With Rofecoxib in a Colorectal Adenoma Chemoprevention Trial,” New England Journal of Medicine 352, no. 11 (2005): 1092–1102, 10.1056/NEJMOA050493. 15713943

[4] J. A. Baron , R. S. Sandler , R. S. Bresalier , et al., “Cardiovascular Events Associated With Rofecoxib: Final Analysis of the APPROVe Trial,” Lancet (London, England) 372, no. 9651 (2008): 1756–1764, 10.1016/S0140-6736(08)61490-7. 18922570

[5] J. Lexchin , “How Safe Are New Drugs? Market Withdrawal of Drugs Approved in Canada Between 1990 and 2009,” Open Medicine 8, no. 1 (2014): e14. 25009681 PMC4085091

[6] Y. Moride , F. Haramburu , A. A. Requejo , and B. Bégaud , “Under‐Reporting of Adverse Drug Reactions in General Practice,” British Journal of Clinical Pharmacology 43, no. 2 (1997): 177–181, 10.1046/j.1365-2125.1997.05417.x. 9131950 PMC2042725

[7] R. Sharrar and G. Dieck , “Monitoring Product Safety in the Postmarketing Environment,” Therapeutic Advances in Drug Safety 4, no. 5 (2013): 211–219. 25114782 10.1177/2042098613490780 PMC4125313

[8] F. Haguinet , A. Bate , and J. U. Stegmann , “The Futility of Adverse Drug Event Reporting Systems for Monitoring Known Safety Issues: A Case Study of Myocardial Infarction With Rofecoxib and Other Drugs,” Pharmacoepidemiology and Drug Safety 33, no. 1 (2024): e5719, 10.1002/PDS.5719. 37867313

[9] A. Bate , K. Hornbuckle , J. Juhaeri , S. P. Motsko , and R. F. Reynolds , “Hypothesis‐Free Signal Detection in Healthcare Databases: Finding Its Value for Pharmacovigilance,” Therapeutic Advances in Drug Safety 10 (2019): 10, 10.1177/2042098619864744. PMC6683315 31428307

[10] M. S. Jansen , O. M. Dekkers , S. le Cessie , et al., “Real‐World Evidence to Inform Regulatory Decision Making: A Scoping Review,” Clinical Pharmacology and Therapeutics 115, no. 6 (2024): 1269–1276, 10.1002/CPT.3218. 38390633

[11] A. Coste , A. Wong , M. Bokern , A. Bate , and I. J. Douglas , “Methods for Drug Safety Signal Detection Using Routinely Collected Observational Electronic Health Care Data: A Systematic Review,” Pharmacoepidemiology and Drug Safety 32, no. 1 (2023): 28–43, 10.1002/PDS.5548. 36218170 PMC10092128

[12] E. Herrett , A. M. Gallagher , K. Bhaskaran , et al., “Data Resource Profile: Clinical Practice Research Datalink (CPRD),” International Journal of Epidemiology 44, no. 3 (2015): 827–836, 10.1093/IJE/DYV098. 26050254 PMC4521131

[13] CPRD GOLD December 2023 dataset|CPRD.

[14] N. F. Khan , S. E. Harrison , and P. W. Rose , “Validity of Diagnostic Coding Within the General Practice Research Database: A Systematic Review,” British Journal of General Practice : The Journal of the Royal College of General Practitioners 60, no. 572 (2010): 199–206, 10.3399/BJGP10X483562. PMC2828861 20202356

[15] A. Arana , A. V. Margulis , C. Varas‐Lorenzo , et al., “Validation of Cardiovascular Outcomes and Risk Factors in the Clinical Practice Research Datalink in the United Kingdom,” Pharmacoepidemiology and Drug Safety 30, no. 2 (2021): 237–237, 10.1002/PDS.5150. 33091194 PMC7821285

[16] E. Bosco , L. Hsueh , K. W. McConeghy , S. Gravenstein , and E. Saade , “Major Adverse Cardiovascular Event Definitions Used in Observational Analysis of Administrative Databases: A Systematic Review,” BMC Medical Research Methodology 21, no. 1 (2021): 241, 10.1186/S12874-021-01440-5. 34742250 PMC8571870

[17] A. Pottegård , J. Hallas , S. V. Wang , and J. J. Gagne , “Identifying Signals of Interest When Screening for Drug‐Outcome Associations in Health Care Data,” British Journal of Clinical Pharmacology 84, no. 9 (2018): 1865–1867, 10.1111/BCP.13634. 29862551 PMC6089829

[18] A. Pottegård , “Core Concepts in Pharmacoepidemiology: Fundamentals of the Cohort and Case–Control Study Designs,” Pharmacoepidemiology and Drug Safety 31, no. 8 (2022): 817–826, 10.1002/pds.5482. 35621007 PMC9545534

[19] The European Network of Centres for Pharmacoepidemiology and Pharmacovigilance (ENCePP) , Guide on Methodological Standards in Pharmacoepidemiology. 2024. EMA/95098/2010.

[20] M. Maclure , “The Case‐Crossover Design: A Method for Studying Transient Effects on the Risk of Acute Events,” American Journal of Epidemiology 133, no. 2 (1991): 144–153, 10.1093/oxfordjournals.aje.a115853. 1985444

[21] D. J. Graham , D. Campen , R. Hui , et al., “Risk of Acute Myocardial Infarction and Sudden Cardiac Death in Patients Treated With Cyclo‐Oxygenase 2 Selective and Non‐Selective Non‐Steroidal Anti‐Inflammatory Drugs: Nested Case‐Control Study,” Lancet 365, no. 9458 (2005): 475–481, 10.1016/S0140-6736(05)70270-1. 15705456

[22] C. Bombardier , L. Laine , A. Reicin , et al., “Comparison of Upper Gastrointestinal Toxicity of Rofecoxib and Naproxen in Patients With Rheumatoid Arthritis,” New England Journal of Medicine 343, no. 21 (2000): 1520–1528, 10.1056/nejm200011233432103. 11087881

[23] S. Abtahi , J. H. M. Driessen , A. M. Burden , et al., “Concomitant Use of Oral Glucocorticoids and Proton Pump Inhibitors and Risk of Osteoporotic Fractures Among Patients With Rheumatoid Arthritis: A Population‐Based Cohort Study,” Annals of the Rheumatic Diseases 80, no. 4 (2021): 423–431, 10.1136/annrheumdis-2020-218758. 33310727

[24] S. Abtahi , J. H. M. Driessen , A. M. Burden , et al., “Low‐Dose Oral Glucocorticoid Therapy and Risk of Osteoporotic Fractures in Patients With Rheumatoid Arthritis: A Cohort Study Using the Clinical Practice Research Datalink,” Rheumatology (Oxford, England) 61, no. 4 (2022): 1448–1458, 10.1093/rheumatology/keab548. 34255815 PMC8996777

[25] O. Aalen , “Nonparametric Inference for a Family of Counting Processes,” Annals of Statistics 6, no. 4 (1978): 701–726.

[26] P. K. Andersen , Ø. Borgan , R. D. Gill , and N. Keiding , “Springer Series in Statistics,” in Statistical Models Based on Counting Processes, vol. XI, 1st ed. (Springer New York, 1993), 784.

[27] P. H. Lee , “Should We Adjust for a Confounder if Empirical and Theoretical Criteria Yield Contradictory Results? A Simulation Study,” Scientific Reports 4, no. 1 (2014): 6085, 10.1038/srep06085. 25124526 PMC5381407

[28] F. Andersohn , S. Suissa , and E. Garbe , “Use of First‐ and Second‐Generation Cyclooxygenase‐2–Selective Nonsteroidal Antiinflammatory Drugs and Risk of Acute Myocardial Infarction,” Circulation 113, no. 16 (2006): 1950–1957, 10.1161/CIRCULATIONAHA.105.602425. 16618816

[29] V. K. Patadia , M. J. Schuemie , P. M. Coloma , et al., “Can Electronic Health Records Databases Complement Spontaneous Reporting System Databases? A Historical‐Reconstruction of the Association of Rofecoxib and Acute Myocardial Infarction,” Frontiers in Pharmacology 9 (2018): 594–594, 10.3389/FPHAR.2018.00594/BIBTEX. 29928230 PMC5997784

[30] S. H. Abbasi , L. C. Lund , J. Hallas , and A. Pottegård , “Sequential Epidemiological Analyses of Real‐World Data: A Tool for Prospective Drug Safety Surveillance From the Rofecoxib Example,” Drug Safety 48, no. 5 (2025): 489–502, 10.1007/s40264-024-01512-7. 39869300 PMC11982122

[31] M. Maclure , B. Fireman , J. C. Nelson , et al., “When Should Case‐Only Designs Be Used for Safety Monitoring of Medical Products?,” Pharmacoepidemiology and Drug Safety 21, no. Suppl 1 (2012): 50–61, 10.1002/pds.2330. 22262593

[32] R. Pannala , A. Basu , G. M. Petersen , and S. T. Chari , “New‐Onset Diabetes: A Potential Clue to the Early Diagnosis of Pancreatic Cancer,” Lancet Oncology 10, no. 1 (2009): 88–95, 10.1016/s1470-2045(08)70337-1. 19111249 PMC2795483

[33] J. A. Delaney and S. Suissa , “The Case‐Crossover Study Design in Pharmacoepidemiology,” Statistical Methods in Medical Research 18, no. 1 (2009): 53–65, 10.1177/0962280208092346. 18765504

