# Supplementary material for: Early Identification of Cardiovascular Adverse Events Associated With Rofecoxib Using Real‐World Data From the UK: A Nested Case–Control and Case‐Crossover Study
Source: Pharmacoepidemiol Drug Saf. 2026 Mar 9;35(3):e70343. doi: 10.1002/pds.70343 (PMC12971294; doi:10.1002/pds.70343)
Supplement: Supplementary file 1 — Table S1: Subgroup analysis in the nested case‐control design of the association between rofecoxib and major cardiovascular adverse events in the UK. [file PDS-35-e70343-s001.docx]

**Early identification of cardiovascular adverse events associated with rofecoxib using real-world data from the UK: A nested case-control and case-crossover study**

Pharmacoepidemiology and Drug Safety journal

Authors:

Donya Moslemzadeh,^1^ Patrick C. Souverein,^1^ Svetlana V. Belitser,^1^ Eibert R. Heerdink,^1,2^ Olaf H. Klungel,^1^ Shahab Abtahi.^1^

Affiliations:

1. Division of Pharmacoepidemiology and Clinical Pharmacology, Utrecht Institute for Pharmaceutical Sciences, Utrecht University, Utrecht, the Netherlands.

2. Research Group Innovations of Pharmaceutical Care, Utrecht University of Applied Sciences, Utrecht, The Netherlands

Corresponding author:

Dr. Shahab Abtahi, Universiteit Utrecht, David de Wiedgebouw, Universiteitsweg 99, 3584 CG Utrecht, the Netherlands, email: s.abtahi@uu.nl

Table S1. Subgroup analysis in the nested case-control design of the association between rofecoxib and major cardiovascular adverse events in the UK.

|  | **Cases** | **Controls** | **Crude IR** | **Adjusted IR^a^** |
| --- | --- | --- | --- | --- |
|  | **(% total)** | **(% total)** | **(95% CI)** | **(95% CI)** |
| **Rofecoxib exposure in Subgroups** |  |  |  |  |
| ***Stratified by sex*** |  |  |  |  |
| Men | 1103 | 3444 |  |  |
| Past use | 493 (45%) | 1756 (51%) | reference | |
| Current use | 480 (44%) | 1310 (38%) | 1.27 (1.09-1.48) | 1.26 (1.07-1.48) |
| Recent use | 130 (12%) | 378 (11%) | 1.22 (0.97-1.53) | 1.18 (0.93-1.50) |
| Women | 2115 | 7301 |  |  |
| Past use | 1040 (49%) | 3776 (52%) | reference | |
| Current use | 870 (41%) | 2715 (37%) | 1.14 (1.03-1.27) | 1.14 (1.02-1.28) |
| Recent use | 205 (10%) | 810 (11%) | 0.91 (0.76-1.08) | 0.91 (0.76-1.09) |
| ***Stratified by age*** |  |  |  |  |
| 18-49 years old | 83 | 271 |  |  |
| Past use | 37 (45%) | 148 (55%) | reference | |
| Current use | 37 (45%) | 82 (30%) | 1.76 (0.99-3.12) | 1.30 (0.67-2.50)^b^ |
| Recent use | 9 (11%) | 41 (15%) | 0.84 (0.36-1.92) | 0.92 (0.40-2.16)^b^ |
| 50-64 years old | 527 | 1951 |  |  |
| Past use | 255 (48%) | 995 (51%) | reference | |
| Current use | 225 (43%) | 742 (38%) | 1.20 (0.97-1.49) | 1.19 (0.93-1.51) |
| Recent use | 47 (9%) | 214 (11%) | 0.87 (0.60-1.26) | 0.82 (0.55-1.21) |
| 65-79 years old | 1506 | 5336 |  |  |
| Past use | 712 (47%) | 2725 (51%) | reference | |
| Current use | 625 (42%) | 2013 (38%) | 1.16 (1.02-1.32) | 1.15 (1.00-1.32) |
| Recent use | 169 (11%) | 598 (11%) | 1.06 (0.87-1.29) | 1.06 (0.87-1.31) |
| 80+ years old | 1102 | 3187 |  |  |
| Past use | 529 (48%) | 1664 (52%) | reference | |
| Current use | 463 (42%) | 1188 (37%) | 1.13 (0.97-1.33) | 1.13 (0.96-1.34) |
| Recent use | 110 (10%) | 335 (11%) | 0.99 (0.77-1.27) | 0.98 (0.75-1.26) |
| ***Stratified by indication*** |  |  |  |  |
| OA | 2271 | 7309 |  |  |
| Past use | 1096 (48%) | 3790 (52%) | reference | |
| Current use | 949 (42%) | 2725 (37%) | 1.19 (1.07-1.33) | 1.19 (1.06-1.34)^c^ |
| Recent use | 226 (10%) | 794 (11%) | 1.02 (0.86-1.22) | 1.02 (0.84-1.22)^c^ |
| Non-OA | 947 | 3436 |  |  |
| Past use | 437 (46%) | 1742 (51%) | reference | |
| Current use | 401 (42%) | 1300 (38%) | 1.08 (0.88-1.32) | 1.04 (0.84-1.30)^c^ |
| Recent use | 109 (12%) | 394 (12%) | 1.00 (0.74-1.37) | 0.95 (0.69-1.33)^c^ |
| RA | 231 | 601 |  |  |
| Past use | 99 (43%) | 301 (50%) | reference | |
| Current use | 114 (49%) | 239 (40%) | 3.15 (1.00-9.87) | 4.32 (0.89-21.04)^c.d^ |
| Recent use | 18 (8%) | 61 (10%) | 1.32 (0.31-5.60) | 3.17 (0.49-20.59)^c,d^ |
| Non-RA | 2987 | 10144 |  |  |
| Past use | 1434 (48%) | 5231 (52%) | reference | |
| Current use | 1236 (41%) | 3786 (37%) | 1.16 (1.06-1.27) | 1.15 (1.05-1.27)^c^ |
| Recent use | 317 (11%) | 1127 (11%) | 1.02 (0.88-1.18) | 1.02 (0.88-1.18)^c^ |
| Back pain | 1659 | 5234 |  |  |
| Past use | 808 (49%) | 2839 (54%) | reference | |
| Current use | 665 (40%) | 1805 (35%) | 1.34 (1.16-1.55) | 1.30 (1.11 - 1.51)^c^ |
| Recent use | 186 (11%) | 590 (11%) | 1.17 (0.94-1.45) | 1.21 (0.96 - 1.52)^c^ |
| No-Back pain | 1559 | 5511 |  |  |
| Past use | 725 (47%) | 2693 (49%) | reference | |
| Current use | 685 (44%) | 2220 (40%) | 1.10 (0.95-1.27) | 1.12 (0.96- 1.30)^c^ |
| Recent use | 149 (10%) | 598 (11%) | 0.85 (0.67-1.08) | 0.81 (0.63-1.04)^c^ |
| ***Stratified by history of CVD*** |  |  |  |  |
| History of CVD | 1395 | 3099 |  |  |
| Past use | 675 (48.4%) | 1624 (52.4%) | reference | |
| Current use | 568 (40.7%) | 1113 (35.9%) | 1.20 (1.00-1.44) | 1.17 (0.96-1.41)^c^ |
| Recent use | 152 (10.9%) | 362 (11.7%) | 1.04 (0.79-1.37) | 0.99 (0.74-1.32)^c^ |
| No history of CVD | 1823 | 7646 |  |  |
| Past use | 858 (47.1%) | 3908 (51.1%) | reference | |
| Current use | 782 (42.9%) | 2912 (38.1) | 1.23 (1.09-1.40) | 1.23 (1.08-1.41)^c^ |
| Recent use | 183 (10.0%) | 826 (10.8%) | 0.92 (0.76-1.13) | 0.91 (0.74-1.12)^c^ |
| *IR: intensity ratio, CI: confidence interval.* | | | | |
| *^a^ Adjusted for smoking status, BMI, alcohol abuse, RA, OA, back pain, migraine, cardiovascular disease,  hyperlipidaemia, hypertension, chronic liver disease, chronic kidney disease, diabetes, malignant neoplasms,  corticosteroids, platelet aggregation inhibitors, NSAIDs and healthcare utilisation.*  *^b^ Adjusted for cardiovascular disease, hyperlipidaemia, hypertension, diabetes, NSAIDs and healthcare utilisation.* | | | | |
| *^c^ Adjusted model excluded the stratifying variable (i.e., OA, RA, backpain, or cardiovascular disease).* | | | | |
| *^d^ Adjusted for smoking status, BMI, cardiovascular disease, hypertension and diabetes.* | | | | |
